# Surgical Treatment for Achalasia of the Esophagus: Laparoscopic Heller Myotomy

**DOI:** 10.1155/2013/708327

**Published:** 2013-11-18

**Authors:** Gonzalo Torres-Villalobos, Luis Alfonso Martin-del-Campo

**Affiliations:** ^1^Department of Surgery, Instituto Nacional de Ciencias Médicas y Nutrición “Salvador Zubirán”, Vasco de Quiroga No. 15, Colonia Seccion XVI, 14000 Tlalpan, México, DF, Mexico; ^2^Experimental Surgery Department, Instituto Nacional de Ciencias Médicas y Nutrición “Salvador Zubirán”, Vasco de Quiroga No. 15, Colonia Seccion XVI, 14000 Tlalpan, México, DF, Mexico

## Abstract

Achalasia is an esophageal motility disorder that leads to dysphagia, chest pain, and weight loss. Its diagnosis is clinically suspected and is confirmed with esophageal manometry. Although pneumatic dilation has a role in the treatment of patients with achalasia, laparoscopic Heller myotomy is considered by many experts as the best treatment modality for most patients with newly diagnosed achalasia. This review will focus on the surgical treatment of achalasia, with special emphasis on laparoscopic Heller myotomy. We will also present a brief discussion of the evaluation of patients with persistent or recurrent symptoms after surgical treatment for achalasia and emerging technologies such as LESS, robot-assisted myotomy, and POEM.

## 1. Introduction

Achalasia is a primary esophageal motility disease that usually presents with progressive dysphagia, chest pain, regurgitation, and weight loss. Its etiology is not fully understood, and its incidence is approximately 1 to 3 cases of 100,000 persons per year [1].

Although achalasia can be suspected using clinical, radiographic, and endoscopic information, definite diagnosis can only be made using esophageal manometry [2], which shows the absence of esophageal motility and in most cases inappropriate lower esophageal sphincter (LES) relaxation. High-resolution manometry can be used to further study esophageal motility in patients with achalasia by categorizing patients into 3 subtypes that can predict patient response to endoscopic or surgical treatment [3].

Treatment for patients with achalasia focuses on symptoms improvement. Endoscopic and surgical approaches for treating achalasia seek to overcome esophageal outflow obstruction while trying to prevent the development of gastroesophageal reflux disease (GERD) and its associated complications [1, 4, 5]. This review will focus on the surgical treatment of achalasia with special emphasis on laparoscopic Heller myotomy.

## 2. Surgical Treatment of Achalasia: Is Laparoscopy Better?

The main objective in surgery for achalasia is the disruption of the LES muscular fibers [6, 7]. The first description of an esophageal myotomy for achalasia was in 1913 by Heller [8], in which both anterior and posterior muscle fibers were divided. A modified procedure currently known as Heller myotomy is the longitudinal division of the anterior muscle fibers and has become the standard surgical approach for achalasia [4]. 

Myotomy can be safely performed using open abdominal and thoracic approaches, and for more than two decades, it has also been done using laparoscopy and thoracoscopy [6]. A meta-analysis by Campos et al. [9] showed that both the thoracic and the abdominal open approaches lead to similar symptom improvement, but the former is associated with twice more GERD symptoms after surgery. When laparoscopy and thoracoscopy are compared, laparoscopy has shown better symptom improvement rates and lower GERD incidence. This difference could be attributed to the fundoplication routinely performed in the laparoscopic group. There are no differences in postoperative complications between these surgical options, but laparoscopy has shown to reduce hospital stay, have less bleeding, have lower analgesic use, and allow for shorter time of return to normal activities. Therefore, laparoscopic myotomy is now considered the surgical procedure of choice for treating achalasia.

## 3. Laparoscopic Myotomy: Surgical Technique

The patient is placed on the operating table in the supine position, and pneumoperitoneum is established followed by placement of 3 to 4 additional trocars using direct visualization.

**Figure 1:**
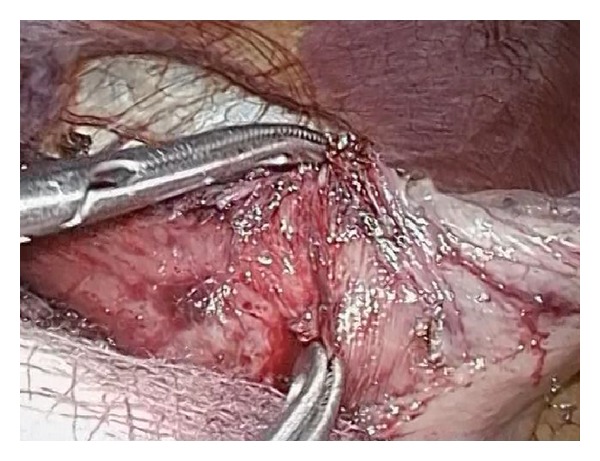
Blunt dissection for myotomy.

Dissection can be started by proximal mobilization of the gastric fundus to prepare for a fundoplication as short vessels are divided using harmonic scalpel. If a hiatal hernia is found, it should be reduced and an adequate intra-abdominal esophagus length must be obtained. Although it has been shown that there may be variations between the endoscopic location of the squamous columnar junction and the LES [10], endoscopy is used to verify completion of the myotomy by identifying a wide open GE junction with no visible crossing residual muscle fibers [6, 11]. The esophagogastric junction (EGJ) is exposed for 6 to 8 cm proximally, and the myotomy is performed longitudinally in the anterior esophageal axis (Figure 1) using blunt dissection, electric hook, scissors, or the harmonic scalpel. Caution must be taken to avoid injury to the esophageal mucosa, especially when using hook cautery or harmonic scalpel because a burned mucosa may perforate in the postoperative period. The procedure is completed by performing a partial anterior (Dor) or a posterior (Toupet) fundoplication.

### 3.1. Myotomy

**Figure 2:**
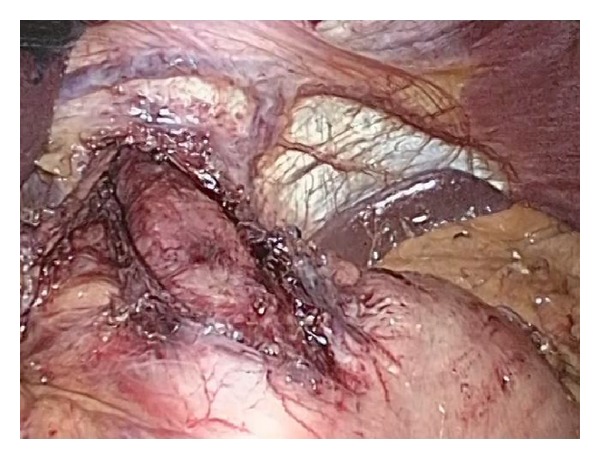
Myotomy extent proximal and distal to the LES.

The best place to start the myotomy is about 2 cm above the gastroesophageal junction, where the submucosal plane is easier to find. Essential to the operation is the length of the myotomy (Figure 2). Some recommendations advocate for 4 to 8 cm proximal and 0.5 to 2 cm distal myotomy [6], which have been associated to lower dysphagia rates and LES resting pressures. Oelschlager et al. have shown that when the distal myotomy in the stomach is increased to 3 cm, both dysphagia rates and LES resting pressures are further reduced, with no associated increase in pyrosis, regurgitation, or thoracic pain [12].

### 3.2. Fundoplication

When a fundoplication is not performed after myotomy, 47% to 100% of patients have postoperative pH-metry confirmed GERD [13, 14]. Some of these patients progress to erosive esophagitis, esophageal stenosis, and Barrett's esophagus [7].

On the other hand, given the total absence of esophageal peristalsis in patients with achalasia, the risk of performing a total fundoplication is to have persistent or recurrent dysphagia [5]. This was demonstrated in a prospective study that compared total (Nissen) versus partial anterior (Dor) fundoplication after Heller myotomy; it showed equal GERD control but higher dysphagia rate (15% versus 2.8%) for the total fundoplication group after 5 years of followup [15]. 

**Figure 3:**
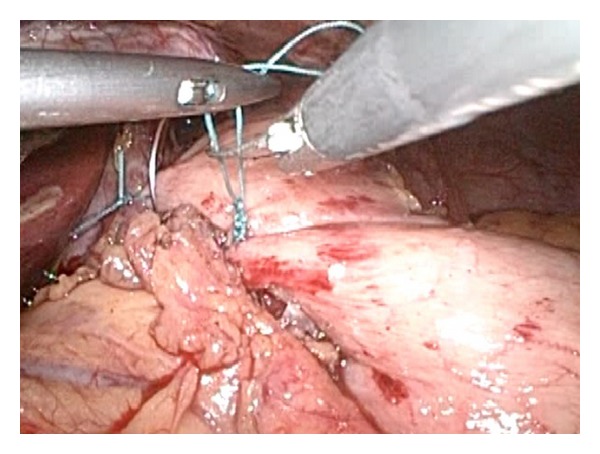
Creation of an anterior partial fundoplication after myotomy.

Therefore, partial fundoplication is the procedure of choice after Heller myotomy (Figure 3). The differences between anterior 180° (Dor) and posterior 270° (Toupet) fundoplication were recently studied in a multicentric, randomized controlled trial that showed no subjective differences in dysphagia or reflux between both groups but did show a higher (not statistically significant) abnormal 24-hour pH-metry (41.7% versus 21%) for the Dor group [16]. Current guidelines state that further high quality is needed in order to find the ideal antireflux procedure after myotomy [6]. We are currently running a prospective, randomized trial comparing Dor versus Toupet fundoplication and evaluating postoperative pH-metry and high-resolution manometry to further solve this question.

## 4. Complications after Surgical Treatment

About 6.3% of the patients who are treated surgically for achalasia have postoperative complications, and only 0.7% of them are clinically relevant [6]. Esophageal perforation may occur in about 7% of the patients [9], but patients who have been previously treated with endoscopic dilation or botulin toxin may have higher perforation rates [6].

Conversion to an open procedure is not frequent in experienced centers and is usually due to esophageal perforation or bleeding. Mortality from laparoscopic Heller myotomy is 0% in most series [4].

## 5. Emerging Technologies

### 5.1. Laparoendoscopic Single Site Surgery (LESS)

LESS is a surgical approach that uses a single port generally placed in the umbilical scar. There are few reports regarding LESS Heller myotomy. A single center experience report of 66 patients showed that a LESS approach can offer similar symptomatic response and patient satisfaction rates to those of the traditional laparoscopic approach, at the cost of longer operative times (117 versus 93 minutes) and the need for extra port placement in 16% of patients [17]. 

The authors of this report conclude that LESS Heller myotomy is feasible, safe, and effective and is cosmetically superior due to a minimal umbilical scar. The same group has recently published its updated experience with the LESS Heller myotomy and concluded that surgeons experienced in the conventional laparoscopic myotomy can quickly attain proficiency with this novel approach [18]. To this date, the only advantage seems to be cosmetic.

### 5.2. Robot-Assisted Myotomy

Among the advantages of robotic surgery are improved dexterity and a high quality three-dimensional view of the procedure [19]. The first published experience of robot-assisted myotomy reported on 54 procedures, with no esophageal perforation and93% of the patients reporting dysphagia improvement after a short-term followup [20].

The largest reported series is a retrospective analysis of 2,683 cases, including 2,116 laparoscopic and 149 robot-assisted myotomies. There were no differences regarding morbidity, mortality, hospital stay, or readmission rates when both approaches were compared. Nevertheless, the robotic approach was associated with a higher cost [21].

### 5.3. Per Oral Endoscopic Myotomy (POEM)

Ortega et al. first described a less invasive endoscopic approach for achalasia in 1980 [22], but it was abandoned for almost 3 decades because of concerns regarding the risk of directly incising esophageal mucosa [23]. Recently, there has been a large interest in modified versions of this technique that are based on the creation of an esophageal submucosal tunnel approximately 13 cm proximal to the EGJ to further create a myotomy of the inner circular esophageal muscle fibers [5]. There have been many variations in the technique, including many different myotomy lengths and circular plus longitudinal fiber myotomy.

When comparing POEM perioperative results against laparoscopic myotomy, a nonrandomized trial showed that POEM is associated with less blood loss (<10 versus 50 ml, *P* < 0.001) and shorter operative time (113 versus 124 min, *P* < 0.05) at the cost of higher pain scores on day 2 with no differences regarding complication rates or length of stay [24]. 

Inoue et al. have performed this procedure more than 100 times in humans and have reported significant improvement in dysphagia and up to 70% reduction in LES resting pressure [25]. A recent report found 82% dysphagia remission after POEM on a 12-month followup [26]. Another nonrandomized prospective study states that POEM is equally effective for dysphagia relief in the short-term followup [27]. Up to 10% of patients who undergo POEM have pneumoperitoneum after the procedure [28]. One of the main concerns of this procedure is that an antireflux procedure cannot be performed, and objectively confirmed GERD rates after POEM are 46% [29].

Although a randomized clinical trial (POEM rcpmt) that will compare POEM versus laparoscopic myotomy is currently recruiting patients, there are currently no high quality evidence and enough followup to endorse POEM as a standard approach. Guidelines for treating achalasia consider POEM as being in its infancy and state that further experience is needed before recommendations can be provided regarding its role in patients with achalasia [6].

## 6. Outcomes after Surgical Treatment

89% (77% to 100%) of patients report symptom improvement after laparoscopic Heller myotomy [9] with satisfaction rates over 90% and associated global improvement in quality of life indicators [6]. Long-term followup (10 years) shows that symptom and quality of life improvement are maintained [30].

Very long-term followup by Csendes et al. shows that failure rates after surgical treatment for achalasia are 7% after 10 years and 35% after 30 years of followup. The authors of this report conclude that this may be due to a progressive increase in esophageal exposure to abnormal gastric reflux, which they demonstrated using pH-metry [31]. 

Risk factors for lower success rates after surgical treatment are severe preoperative dysphagia, low LES pressures, severe esophageal dilation, and previous endoscopic dilation or botulin toxin treatment [6]. Using high-resolution manometry Pandolfino et al. created a new system that classifies achalasia in three types [3]. This system has shown to predict the success rates for patients undergoing Heller myotomy, being 85.4% for type I, 95.3% for type II, and 69.4% for type III [32].

It is known that patients with achalasia have higher esophageal cancer incidence [33, 34], and this risk remains even after surgical treatment [35]. Nevertheless, there are no differences in survival between patients who have received surgical treatment for achalasia and the general population [36], and since 400 endoscopies must be performed to detect 1 cancer, there are currently no recommendations regarding the endoscopic surveillance after Heller myotomy [2].

## 7. Evaluation and Treatment of the Patient with Persistent or Recurrent Symptoms

The most common causes of failure after surgery are incomplete myotomy (33%) which is more frequently found in the gastric myotomy, myotomy fibrosis (27%), fundoplication disruption (13%), tight fundoplication (7%), and a combination of fibrosis and incomplete myotomy (20%) [9]. Outflow obstruction, esophageal dilation, or sigmoid esophagus must be ruled out using an esophagogram if dysphagia recurs. Endoscopy can also show stenosis and obstructive lesions. Manometry could show a persistently hypertensive LES [4].

Approximately 5% of patients will need another intervention [37]. There are multiple reports [38, 39] that support endoscopic dilation or reoperation as both safe and effective options that can improve symptoms and avoid esophagectomy. The Yokohama group reported on the use of POEM in 10 patients with persistent or recurrent dysphagia after Heller myotomy or pneumatic dilation and showed symptomatic improvement and lower LES resting pressures in the short-term followup [40]. Only a minority of patients who show massive esophageal dilation will be candidates for esophagectomy [41].

## 8. Conclusions

Medical treatment for achalasia is mainly reserved for patients with very high surgical risk given its low long-term success for improving symptoms [42]. Although there are randomized controlled trials [43] that have proven the safety and efficacy of endoscopic dilation for achalasia, evidence ranging from expert opinion to meta-analysis supports laparoscopic Heller myotomy as the best initial treatment for most patients with achalasia [44, 45].

Laparoscopy has steadily positioned as the surgical approach of choice to treat achalasia given its lower hospital stay, less bleeding, less analgesic use, and shorter time of return to normal activities when compared with open approaches. Critical aspects of the laparoscopic Heller myotomy include obtaining an adequate length of intra-abdominal esophagus, identification of the EGJ in order to perform a complete proximal (5 cm) and distal (3 cm) myotomy, and creation of a partial fundoplication.

Approximately 90% of patients will have symptom improvement with Heller myotomy, and the majority of them have no recurrence even after very long-term followup. Nevertheless, a minority of cases will have persistent or recurrent symptoms, and one should thoroughly evaluate these patients in order to adequately provide interventions such as pneumatic dilation or reoperation while reserving esophagectomy for patients with very severe esophageal dilation.

Although technologies such as LESS and POEM are emerging, their use is still under investigation, and there are currently no recommendations that support their use outside the research context.

## References

[1] Gockel I, Sgourakis G, Drescher DG, Lang H (2011). Impact of minimally invasive surgery in the spectrum of current achalasia treatment options. *Scandinavian Journal of Surgery*.

[2] Vaezi MF, Pandolfino JE, Vela MF (2013). ACG clinical guideline: diagnosis and management of achalasia. *American Journal of Gastroenterology*.

[3] Pandolfino JE, Kwiatek MA, Nealis T, Bulsiewicz W, Post J, Kahrilas PJ (2008). Achalasia: a new clinically relevant classification by high-resolution manometry. *Gastroenterology*.

[4] Williams VA, Peters JH (2009). Achalasia of the esophagus: a surgical disease. *Journal of the American College of Surgeons*.

[5] Bello B, Herbella FA, Patti MG (2011). Evolution of the minimally invasive treatment of esophageal achalasia. *World Journal of Surgery*.

[6] Stefanidis D, Richardson W, Farrell TM, Kohn GP, Augenstein V, Fanelli RD (2012). SAGES guidelines for the surgical treatment of esophageal achalasia. *Surgical Endoscopy*.

[7] Francis DL, Katzka DA (2010). Achalasia: update on the disease and its treatment. *Gastroenterology*.

[8] Heller E (1913). Extramukose cardioplastik bein chronischen Cardiospasmus mit Dilatation des Oesophagus. *Mitteilungen aus Den Grenzgebieten Der Medizin Und Chirurgie*.

[9] Campos GM, Vittinghoff E, Rabl C (2009). Endoscopic and surgical treatments for achalasia: a systematic review and meta-analysis. *Annals of Surgery*.

[10] Csendes A, Maluenda F, Braghetto I, Csendes P, Henriquez A, Quesada MS (1993). Location of the lower oesophageal sphincter and the squamous columnar mucosal junction in 109 healthy controls and 778 patients with different degrees of endoscopic oesophagitis. *Gut*.

[11] Bloomston M, Brady P, Rosemurgy AS (2002). Videoscopic Heller myotomy with intraoperative endoscopy promotes optimal outcomes. *Journal of the Society of Laparoendoscopic Surgeons/Society of Laparoendoscopic Surgeons*.

[12] Oelschlager BK, Chang L, Pellegrini CA (2003). Improved outcome after extended gastric myotomy for achalasia. *Archives of Surgery*.

[13] Falkenback D, Johansson J, Öberg S (2003). Heller’s esophagomyotomy with or without a 360° floppy Nissen fundoplication for achalasia. Long-term results from a prospective randomized study. *Diseases of the Esophagus*.

[14] Richards WO, Torquati A, Holzman MD (2004). Heller myotomy versus heller myotomy with dor fundoplication for achalasia: a prospective randomized double-blind clinical trial. *Annals of Surgery*.

[15] Rebecchi F, Giaccone C, Farinella E, Campaci R, Morino M (2008). Randomized controlled trial of laparoscopic heller myotomy plus dor fundoplication versus nissen fundoplication for achalasia long-term results. *Annals of Surgery*.

[16] Rawlings A, Soper NJ, Oelschlager B (2012). Laparoscopic Dor versus Toupet fundoplication following Heller myotomy for achalasia: results of a multicenter, prospective, randomized-controlled trial. *Surgical Endoscopy*.

[17] Barry L, Ross S, Dahal S (2011). Laparoendoscopic single-site Heller myotomy with anterior fundoplication for achalasia. *Surgical Endoscopy*.

[18] Ross SB, Luberice K, Kurian TJ, Paul H, Rosemurgy AS (2013). Defining the learning curve of laparoendoscopic single-site Heller myotomy. *American Journal of Surgery*.

[19] Undre S, Moorthy K, Munz Y (2004). Robot-assisted laparoscopic Heller cardiomyotomy: preliminary UK results. *Digestive Surgery*.

[20] Galvani C, Gorodner MV, Moser F, Baptista M, Donahue P, Horgan S (2006). Laparoscopic Heller myotomy for achalasia facilitated by robotic assistance. *Surgical Endoscopy*.

[21] Shaligram A, Unnirevi J, Simorov A, Kothari VM, Oleynikov D (2012). How does the robot affect outcomes? A retrospective review of open, laparoscopic, and robotic Heller myotomy for achalasia. *Surgical Endoscopy*.

[22] Ortega JA, Madureri V, Perez L (1980). Endoscopic myotomy in the treatment of achalasia. *Gastrointestinal Endoscopy*.

[23] Inoue H, Tianle KM, Ikeda H (2011). Peroral endoscopic myotomy for esophageal achalasia: technique, indication, and outcomes. *Thoracic Surgery Clinics*.

[24] Hungness ES, Teitelbaum EN, Santos BF (2013). Comparison of perioperative outcomes between peroral esophageal myotomy (POEM) and laparoscopic Heller myotomy. *Journal of Gastrointestinal Surgery*.

[25] Inoue H, Minami H, Kobayashi Y (2010). Peroral endoscopic myotomy (POEM) for esophageal achalasia. *Endoscopy*.

[26] Von Renteln D, Fuchs KH, Fockens P (2013). Peroral endoscopic myotomy for the treatment of achalasia: an international prospective multicenter study. *Gastroenterology*.

[27] Ujiki MB, Yetasook AK, Zapf M, Linn JG, Carbray JM, Denham W (2013). Peroral endoscopic myotomy: a short-term comparison with the standard laparoscopic approach. *Surgery*.

[28] Swanström LL, Rieder E, Dunst CM (2011). A stepwise approach and early clinical experience in peroral endoscopic myotomy for the treatment of achalasia and esophageal motility disorders. *Journal of the American College of Surgeons*.

[29] Swanstrom LL, Kurian A, Dunst CM, Sharata A, Bhayani N, Rieder E (2012). Long-term outcomes of an endoscopic myotomy for achalasia: the POEM procedure. *Annals of Surgery*.

[30] Jeansonne LO, White BC, Pilger KE (2007). Ten-year follow-up of laparoscopic Heller myotomy for achalasia shows durability. *Surgical Endoscopy*.

[31] Csendes A, Braghetto I, Burdiles P, Korn O, Csendes P, Henriquez A (2006). Very late results of esophagomyotomy for patients with achalasia: clinical, endoscopic, histologic, manometric, and acid reflux studies in 67 patients for a mean follow-up of 190 months. *Annals of Surgery*.

[32] Salvador R, Costantini M, Zaninotto G (2010). The preoperative manometric pattern predicts the outcome of surgical treatment for esophageal achalasia. *Journal of Gastrointestinal Surgery*.

[33] Zendehdel K, Nyrén O, Edberg A, Ye W (2011). Risk of esophageal adenocarcinoma in achalasia patients, a retrospective cohort study in Sweden. *American Journal of Gastroenterology*.

[34] Leeuwenburgh I, Scholten P, Alderliesten J (2010). Long-term esophageal cancer risk in patients with primary achalasia: a prospective study. *American Journal of Gastroenterology*.

[35] Zaninotto G, Rizzetto C, Zambon P, Guzzinati S, Finotti E, Costantini M (2008). Long-term outcome and risk of oesophageal cancer after surgery for achalasia. *British Journal of Surgery*.

[36] Eckardt VF, Hoischen T, Bernhard G (2008). Life expectancy, complications, and causes of death in patients with achalasia: results of a 33-year follow-up investigation. *European Journal of Gastroenterology and Hepatology*.

[37] Bessell JR, Lally CJ, Schloithe A, Jamieson GG, Devitt PG, Watson DI (2006). Laparoscopic cardiomyotomy for achalasia: long-term outcomes. *ANZ Journal of Surgery*.

[38] Loviscek MF, Wright AS, Hinojosa MW (2013). Recurrent dysphagia after Heller myotomy: is esophagectomy always the answer?. *Journal of the American College of Surgeons*.

[39] Petersen RP, Pellegrini CA (2010). Revisional surgery after heller myotomy for esophageal achalasia. *Surgical Laparoscopy, Endoscopy and Percutaneous Techniques*.

[40] Onimaru M, Inoue H, Ikeda H (2013). Peroral endoscopic myotomy is a viable option for failed surgical esophagocardiomyotomy instead of redo surgical heller myotomy: a single center prospective study. *Journal of the American College of Surgeons*.

[41] Molena D, Yang SC (2012). Surgical management of end-stage achalasia. *Seminars in Thoracic and Cardiovascular Surgery*.

[42] Boeckxstaens GE, Zaninotto G, Richter JE (2013). Achalasia. *The Lancet*.

[43] Boeckxstaens GE, Annese V, Des Varannes SB (2011). Pneumatic dilation versus laparoscopic heller’s myotomy for idiopathic achalasia. *The New England Journal of Medicine*.

[44] Patti MG, Pellegrini CA (2012). Esophageal achalasia 2011: pneumatic dilatation or laparoscopic myotomy?. *Journal of Gastrointestinal Surgery*.

[45] Yaghoobi M, Mayrand S, Martel M, Roshan-Afshar I, Bijarchi R, Barkun A (2013). Laparoscopic Heller's myotomy versus pneumatic dilation in the treatment of idiopathic achalasia: a meta-analysis of randomized, controlled trials. *Gastrointestinal Endoscopy*.

